# Evaluating Ozonated Olive Oil and Chlorhexidine Gel as a Therapeutic Intervention for Periodontitis Stage II Grade A

**DOI:** 10.7759/cureus.85001

**Published:** 2025-05-28

**Authors:** Maitri S Patel, Gaurav A Girdhar, Aseem Sharma, Prasanta Majumder, Anwesha Bhattacharjee, Mohd. Shabankhan H Pathan, Shruti D Vyas, Mainul Haque, Santosh Kumar

**Affiliations:** 1 Department of Periodontology and Implantology, Karnavati School of Dentistry, Karnavati University, Gandhinagar, IND; 2 Department of Orthodontics and Dentofacial Orthopaedics, Himachal Institute of Dental Sciences, Paonta Sahib, IND; 3 Department of Public Health Dentistry, Agartala Government Dental College and IGM Hospital, Agartala, IND; 4 Department of Oral Medicine and Radiology, Agartala Government Dental College and IGM Hospital, Agartala, IND; 5 Department of Research, Karnavati School of Dentistry, Karnavati University, Gandhinagar, IND

**Keywords:** antimicrobial treatment, clinical parameters, comparative analysis, gingival health, inflammatory response, oral microbiota, oxidative therapy, periodontal healing, plaque control, pocket depth reduction

## Abstract

Introduction

Periodontal therapy aims to eliminate harmful microorganisms from the gingival sulcus. Still, factors like deep pockets, tooth anatomy, and tissue-invading bacteria can reduce the effectiveness of scaling and root planing (SRP). Antimicrobial agents are often used to enhance SRP outcomes. While chlorhexidine (CHX) is commonly used, its limitations have prompted the exploration of alternatives like ozone therapy. Ozonated olive oil has shown potential therapeutic benefits. This study compares the effectiveness of CHX and ozonated olive oil as adjuncts to SRP in treating periodontitis stage II grade A.

Materials and methods

A longitudinal study with 20 participants was conducted. The control group received SRP with CHX, while the test group received SRP with ozonated olive oil. Clinical parameters, including probing depth, attachment loss, and sulcus bleeding index, were measured at baseline and after three months. Data were analyzed using the Student t-test.

Results

Both groups showed significant improvements in clinical parameters, with no significant differences.

Conclusion

Ozonated olive oil may enhance SRP outcomes in periodontal disease treatment and could be a valuable adjunct. Further studies are needed to confirm its long-term efficacy.

## Introduction

Periodontitis is a chronic inflammatory condition that damages the supporting structures of the teeth. The 2017 classification by the American Academy of Periodontology (AAP) replaced the previous terms “chronic” and “aggressive” with a staging and grading system, categorizing the disease based on its severity, complexity, and risk of progression. This novel system categorizes periodontitis based on its severity, complexity, and risk of progression, using stages (I-IV) and grades [1]. Failure to address aggressive periodontitis (AP) results in a gradual deterioration of these structures, manifesting as gingival bleeding, probing pocket depth (PPD), clinical attachment loss (CAL), relative attachment level (RAL), and alveolar bone resorption, culminating in tooth loss [2].

The organized collection of bacteria known as dental biofilm, which adheres to teeth and is encased in a matrix outside the cells, is the leading cause of periodontitis. Well-established risk factors include smoking and diabetes, while contributing factors encompass occlusal trauma, open interproximal contacts, poorly contoured restorations, mucogingival deformities, and anatomical variations [3]. Based on information from the Global Burden of Disease (GBD) database, there were approximately 1.1 billion cases of severe periodontitis worldwide in 2019. Moreover, there was an 8.44% rise in the age-standardized prevalence rate of severe periodontitis between 1990 and 2019 [4].

Non-surgical periodontal therapy typically includes scaling and root planing (SRP), a standard treatment in periodontics known for its effectiveness in reducing inflammation, decreasing PPD, and improving RAL [5]. This method entails a rigorous therapeutic protocol with inherent constraints, primarily associated with reaching deep periodontal pockets and furcation areas and eradicating specific pathogens. Additionally, academic studies have extensively reported negative results such as receding gums, loss of tooth enamel, and sensitive teeth. Various supplementary treatments have been suggested to address these constraints, primarily involving the administration of systemic or localized antimicrobial substances [6]. While demonstrating efficacy, systemic antimicrobial therapy necessitates a relatively elevated dosage repeatedly over an extended duration to attain the essential inhibitory levels in the sulcular fluid. The supplementary application of local drug administration could offer a favorable outcome, particularly in targeted sites where traditional treatment modalities may prove ineffective [7].

Chlorhexidine (CHX) continues to be recognized as a highly potent local antimicrobial agent, extensively employed for addressing periodontitis at a regional level. Due to its interaction with the negatively charged bacterial cell surface, the cationic CHX molecule exhibits strong antibacterial effectiveness within the periodontal pocket while exhibiting minimal toxicity, patient non-compliance, and the development of resistant microorganisms [8]. Using CHX as a mouth rinse or topical oral gel can lead to specific side effects, including dry mouth (xerostomia), changes in taste perception (hypogeusia), particularly with salt and bitter flavors, and a tongue that may appear discolored or coated. Less frequent adverse events encompass burning sensations (glossodynia), exfoliation of the oral mucosa, enlargement of the parotid gland, and tingling sensations in the mouth. Nevertheless, tooth discoloration is likely the primary undesirable consequence discouraging individuals from utilizing CHX mouthwash [9]. The limitations of CHX drive us to search for a new antimicrobial treatment with fewer adverse effects [1].

There is a growing interest in ozone therapy as a contemporary non-invasive therapeutic approach. This methodology harnesses the potent oxidizing properties of ozone, exhibiting significant antimicrobial efficacy against oral pathogens. Interestingly, no instances of resistance emergence have been documented in the context of gaseous ozone and its aqueous form [10]. Ozone is being explored in dentistry as a potential substitute antiseptic. It possesses potent antimicrobial characteristics against oral pathogens. Ozone in both gaseous and aqueous forms shows effectiveness. Ozone gas can reduce oral cell viability. Aqueous ozone is biocompatible with various oral cells, indicating potential use against oral diseases [11]. Ozone is a naturally formed compound composed of three oxygen atoms. It is a potent antimicrobial agent that destroys bacterial and fungal cell walls and cytoplasmic membranes [12]. Ozone is the allotrope of oxygen. An ozone molecule comprises three oxygen atoms rather than two, rendering it exceptionally reactive for oxidation processes. The heightened oxidation capacity proves essential for eradicating microorganisms, particularly anaerobic ones [1].

Multiple clinical studies found that ozonated oil can improve gum health. Despite in vitro studies indicating ozone's superior antimicrobial effectiveness over CHX, there is a scarcity of literature supporting its clinical application [13-15].

The problem statement of the current study

According to the AAP classification, periodontitis is a common inflammatory condition treated with SRP, often supplemented with CHX [16,17]. However, CHX has drawbacks, including side effects that reduce patient compliance. The search for alternative adjunctive therapies has led to interest in ozone-based treatments. With over a billion cases of severe periodontitis globally, identifying safer and equally effective alternatives to CHX is critical. Exploring ozone-based therapies could address the unmet needs in periodontal care, offering patients an effective treatment option with fewer side effects.

Objective of the study

The present study aims to evaluate and compare the effectiveness of ozonated olive oil and CHX as local drug delivery agents, used adjunctively with SRP, in the non-surgical management of periodontitis stage II grade A. Specifically, the study assesses their impact on the reduction of PPD, thereby determining the comparative clinical efficacy of ozonated olive oil versus CHX in patients with periodontitis stage II grade A [1].

## Materials and methods

The present study was conducted in our institute's Department of Periodontology. The institutional review board (IRB) of Karnavati School of Dentistry, Karnavati University, Gandhi Nagar, India, reviewed and approved the study on May 13, 2024, with Reference No.: KSDEC/23-24/Apr/0011. This study followed the guidelines set out in the 2021 Declaration of Helsinki. The sample size formula is n_0_ = Z^2^δ^2^/e^2^, where n_0_ is the sample size, Z is the standard normal deviation, e is the desired level of precision, and σ^2^ is the variance of an attribute in the population. A longitudinal study was carried out, and 20 participants were chosen to participate in the study based on specific criteria.

Inclusion criteria

A total of 20 patients, 18-65 years of age, were diagnosed with AP according to the AAP [18] with the presence of at least one site in each quadrant with PPD ≥ 5 mm, RAL of ≥3 mm [19], possessing a minimum of 20 teeth in the oral cavity.

Exclusion criteria

Participants with recent antimicrobial therapy within the past six months; individuals with systemic illnesses; previous history of dental treatment or mouthwash use; patients with acute necrotizing ulcerative gingivitis, acute herpetic gingivostomatitis, gingivitis associated with skin conditions, endocrine-metabolic disturbances, hematologic-immunologic disturbances, and drug-induced gingival enlargement or gingival tumors; pregnant or lactating women; and smokers were not included in the study. Twenty qualified individuals who met the requirements and were willing to participate in this research were listed. Before enrollment, patients were provided with comprehensive details about the research protocol, and both verbal consent and written consent were obtained.

Initially, participants underwent thorough full-mouth SRP using an ultrasonic scaler; patients were counseled to use the modified Bass technique and non-medicated toothpaste post-operatively. Subsequently, patients were summoned for the planned intervention on the baseline day. Each participant was subjected to two treatment regimens, targeting sites with PPD ≥ 5 mm and RAL ≥ 3 mm.

The allocation of treatment sites was randomized using a computer-generated simple randomization method with the help of a random number table generated in Microsoft Excel (Microsoft Corp., Redmond, WA, US). This process ensured equal and unbiased distribution of participants into two treatment groups. The test group’s sites received treatment involving SRP, followed by the application of 1 mL of ozonated olive oil. The ozonated oil was prepared by bubbling medical-grade ozone gas through pharmaceutical-grade olive oil until a gel-like consistency was achieved, ensuring optimal saturation and stability. The ozonated olive oil was delivered into the periodontal pockets using a blunt-tipped syringe, ensuring thorough and localized subgingival placement. The test group consisted of five male and five female patients. In contrast, the control group’s sites underwent SRP followed by the application of 1 mL of 1.0% CHX gluconate gel, which was prepared using standard pharmaceutical formulation techniques to achieve a uniform and clinically effective concentration. The CHX gel was similarly administered using a blunt-tipped syringe and gently deposited into the periodontal pockets to ensure adequate subgingival delivery. The control group included four male and six female patients.

Preparation of the stent and application of drugs

Alginate impressions were taken, and dental stone casts were fabricated. These casts were utilized to create occlusal stents for specific sites using self-cure clear acrylic. These stents were employed as a reference for measuring the RAL.

Outcome variables

To minimize interobserver bias, all the clinical parameters were recorded by a single examiner using a calibrated periodontal probe (UNC-15 graduated periodontal probe, Hu-Friedy, Chicago, IL, US). The outcome variables of the study were as follows: (1) PPD, (2) RAL, (3) sulcus bleeding index (SBI) [20], and (4) plaque index (PI) (Löe) [21].

Statistical analysis

**Figure 1:**
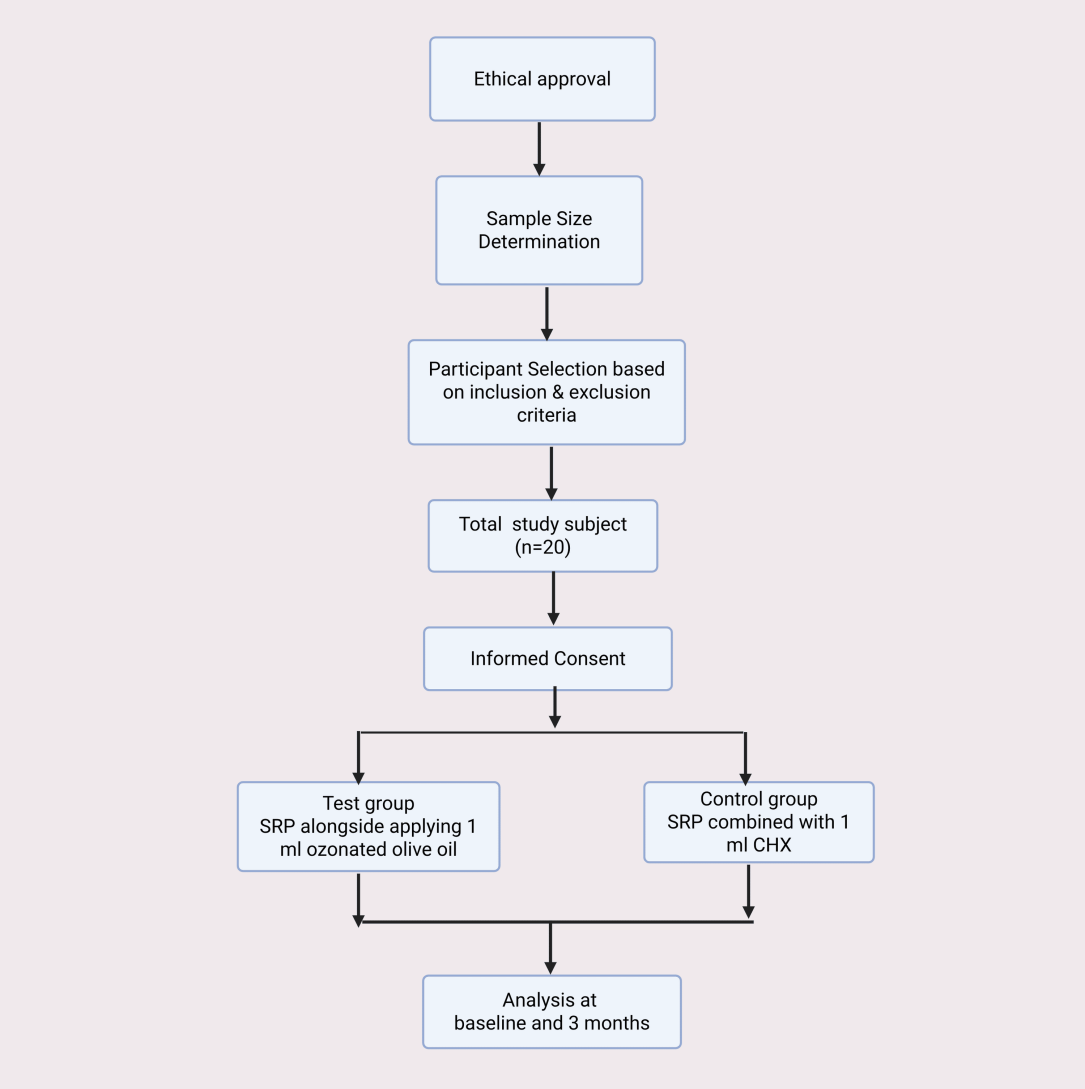
The methodology of this paper. This figure was drawn using the premium version of BioRender, https://BioRender.com x4dlh7g, accessed on May 2, 2025, which has agreement number SJ287REVFX [22]. Image Credit: Maitri S. Patel. SRP: scaling and root planing; CHX: chlorhexidine

The mean differences in clinical parameters between the test and control sites at baseline and three months were compared using an independent sample t-test. Within-group differences from baseline to three months were analyzed using repeated measures ANOVA. All statistical analyses were performed in STATA (version 15) (StataCorp LLC, College Station, TX, US), and graphical charts were created using GraphPad Prism 10.4.0 (Dotmatics, Boston, MA, US). A p-value of ≤0.05 was considered statistically significant. The methodology of this paper is illustrated in Figure 1.

## Results

**Figure 2:**
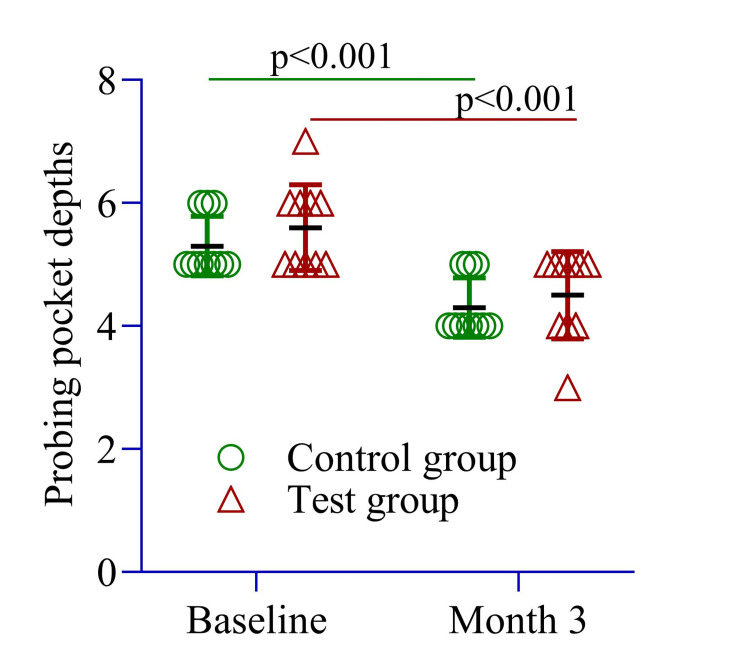
The mean difference in probing pocket depth was compared between the control and test groups at baseline and three months and within each group from baseline to three months. An independent sample t-test was conducted to assess differences between the groups, while repeated measures ANOVA was used to evaluate within-group changes over time. Image Credit: Md. Ahsanul Haq.

Figure 2 compares mean differences in clinical parameters between the test and control sites at baseline. An independent sample t-test revealed no statistically significant differences between the groups (p > 0.05 for all parameters). The test site had a slightly higher mean PPD (5.60 ± 0.69 mm) than the control site (5.30 ± 0.48 mm, p = 0.27). Additionally, within-group differences were assessed using repeated measures ANOVA. In the control group, PPD significantly decreased from 5.30 ± 0.48 mm at baseline to 4.30 ± 0.48 mm at three months (p < 0.001). Similarly, in the test group, PPD decreased from 5.60 ± 0.69 mm at baseline to 4.50 ± 0.70 mm at three months (p = 0.003).

**Figure 3:**
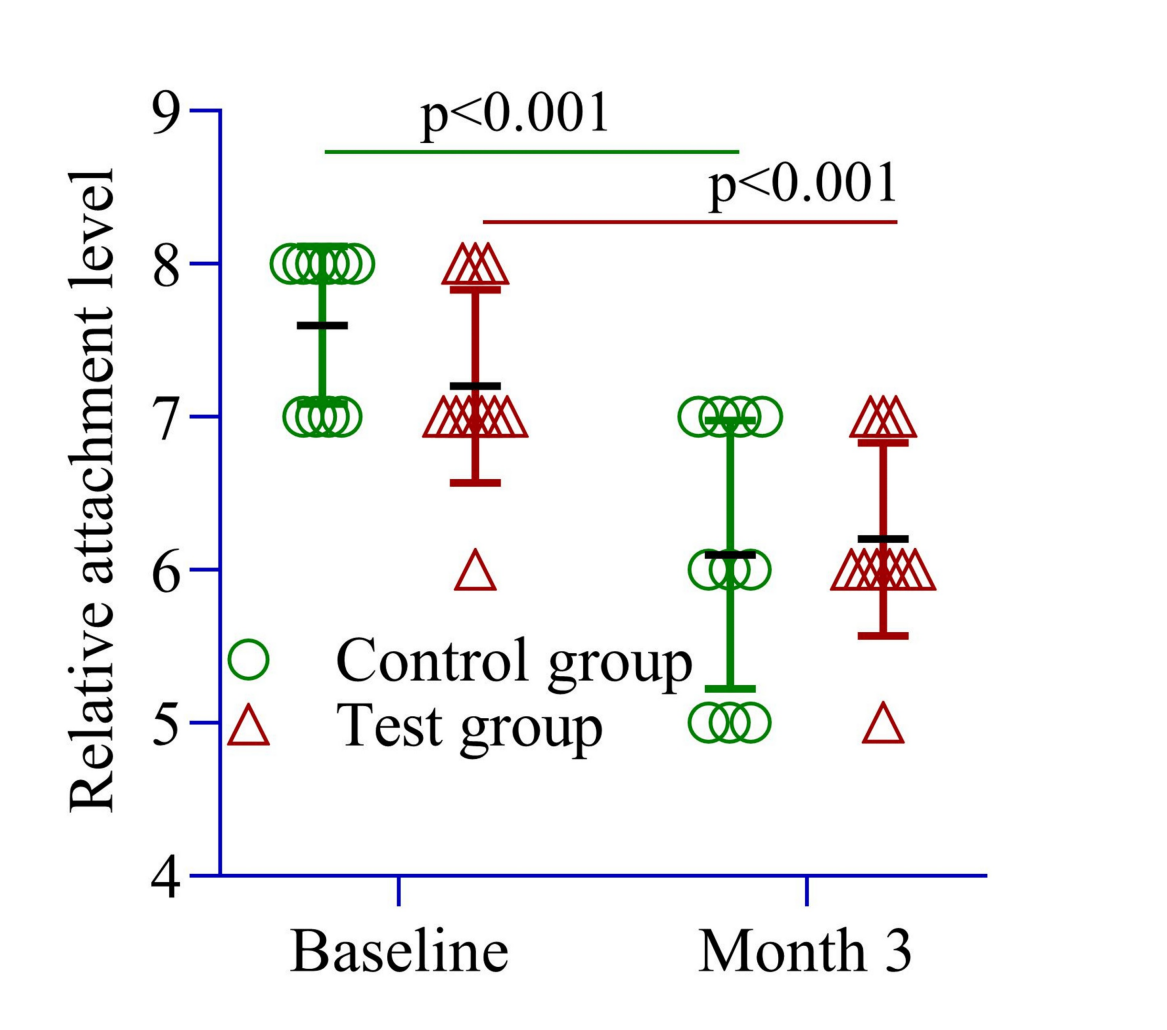
The mean difference in relative attachment level was compared between the control and test groups at baseline and three months and within each group from baseline to three months. An independent sample t-test was conducted to assess differences between the groups, while repeated measures ANOVA was used to evaluate within-group changes over time. Image Credit: Md. Ahsanul Haq.

The RAL was slightly lower in the test group (7.20 ± 0.63 mm) compared to the control group (7.60 ± 0.51 mm, p = 0.13). Within-group analysis showed a significant improvement in the control group, with the RAL decreasing from 7.60 ± 0.51 mm at baseline to 6.10 ± 0.87 mm at three months (Figure 3). Similarly, in the test group, the RAL improved from 7.20 ± 0.63 mm to 6.20 ± 0.63 mm (p = 0.002) over the same period (Figure 3).

**Figure 4:**
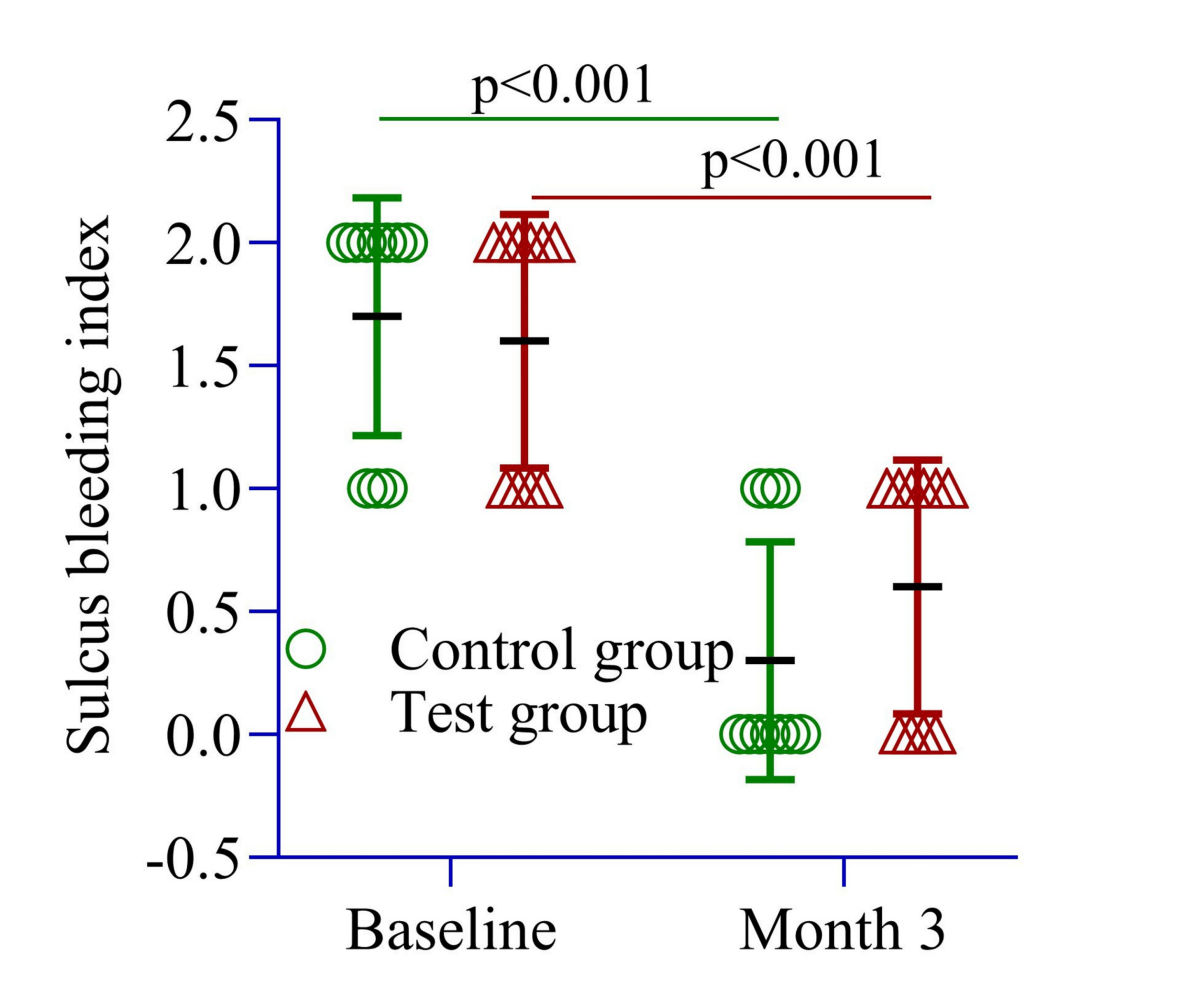
The mean difference in the sulcus bleeding index was compared between the control and test groups at three months and within each group from baseline to three months. An independent sample t-test was conducted to assess differences between the groups, while repeated measures ANOVA was used to evaluate within-group changes over time. Image Credit: Md. Ahsanul Haq.

The SBI was similar between the control and test groups, with values of 1.60 ± 0.51 and 1.70 ± 0.48, respectively (p = 0.661) (Figure 4). Within-group analysis showed a significant reduction in the control group, decreasing from 1.70 ± 0.48 at baseline to 0.30 ± 0.48 at three months, indicating reduced inflammation. Similarly, the test group exhibited a significant decrease in the SBI from 1.60 ± 0.51 to 0.60 ± 0.51 (p = 0.001) over the same period (Figure 4).

**Figure 5:**
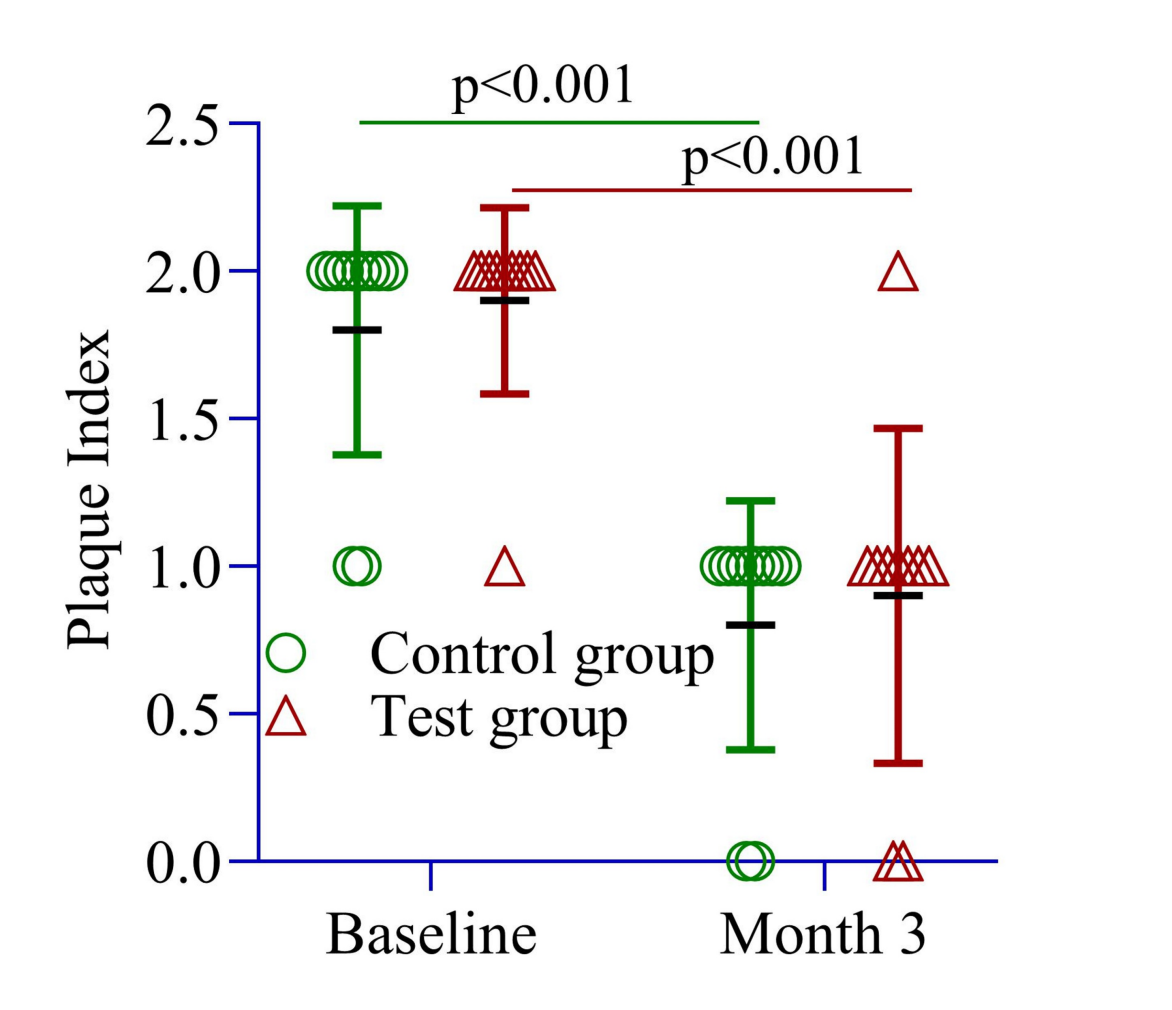
The mean difference in plaque index was compared between the control and test groups at baseline and three months and within each group from baseline to three months. An independent sample t-test was conducted to assess differences between the groups, while repeated measures ANOVA was used to evaluate within-group changes over time. Image Credit: Md. Ahsanul Haq.

The PI between the test and control groups was 1.90 ± 0.31 and 1.80 ± 0.42 (p = 0.55), respectively. These findings indicate that both groups had similar baseline clinical characteristics (Figure 5). Additionally, the PI dropped significantly from 1.80 ± 0.42 to 0.80 ± 0.42 in the control group from baseline to month 3, reflecting improved oral hygiene (Figure 5). Similarly, the test group showed a similar decrease from 1.90 ± 0.31 to 0.90 ± 0.56 from baseline to month 3 (p = 0.001), indicating better oral health outcomes (Figure 5).

PPD reduction was slightly greater in the test site (1.10 ± 0.01 mm) compared to the control site (1.00 ± 0.0 mm, p = 0.10) (Figure 2). The RAL change was 1.0 ± 0.0 mm in the test site and 1.5 ± 0.26 mm in the control site (p = 0.23) (Figure 3). The SBI improvement was comparable between groups (1.0 ± 0.0 vs. 1.40 ± 0.0, p = 0.54) (Figure 4), while the PI remained similar (1.0 ± 0.25 vs. 1.0 ± 0.0, p = 0.22) (Figure 5). These findings suggest that both groups experienced similar clinical outcomes post-treatment, with no significant differences.

## Discussion

Both the test and control groups demonstrated improvements in clinical parameters, including PPD, RAL, SBI, and PI, after three months of treatment. These improvements were observed within each group, indicating positive changes. However, no significant differences were found in the clinical parameters when comparing the test and control groups at three months. This suggests that while both treatments were effective, there was no clear advantage of one over the other.

Historically, the mainstay of periodontal treatment is the removal of plaque and calculus through manual means, along with the additional use of antibiotics and antiseptics. However, the robust antimicrobial properties of ozone, combined with its ability to modulate the immune system's response, suggest it is a promising therapeutic intervention for tackling the multifaceted aspects of periodontal disease [23].

Adjunctive antimicrobials can be administered systemically or locally for individuals with localized pockets or non-responsive and recurrent sites. Localized application is preferred due to its lower occurrence of adverse effects, reduced likelihood of bacterial resistance development, and improved patient adherence compared to systemic antimicrobial use [24].

**Figure 6:**
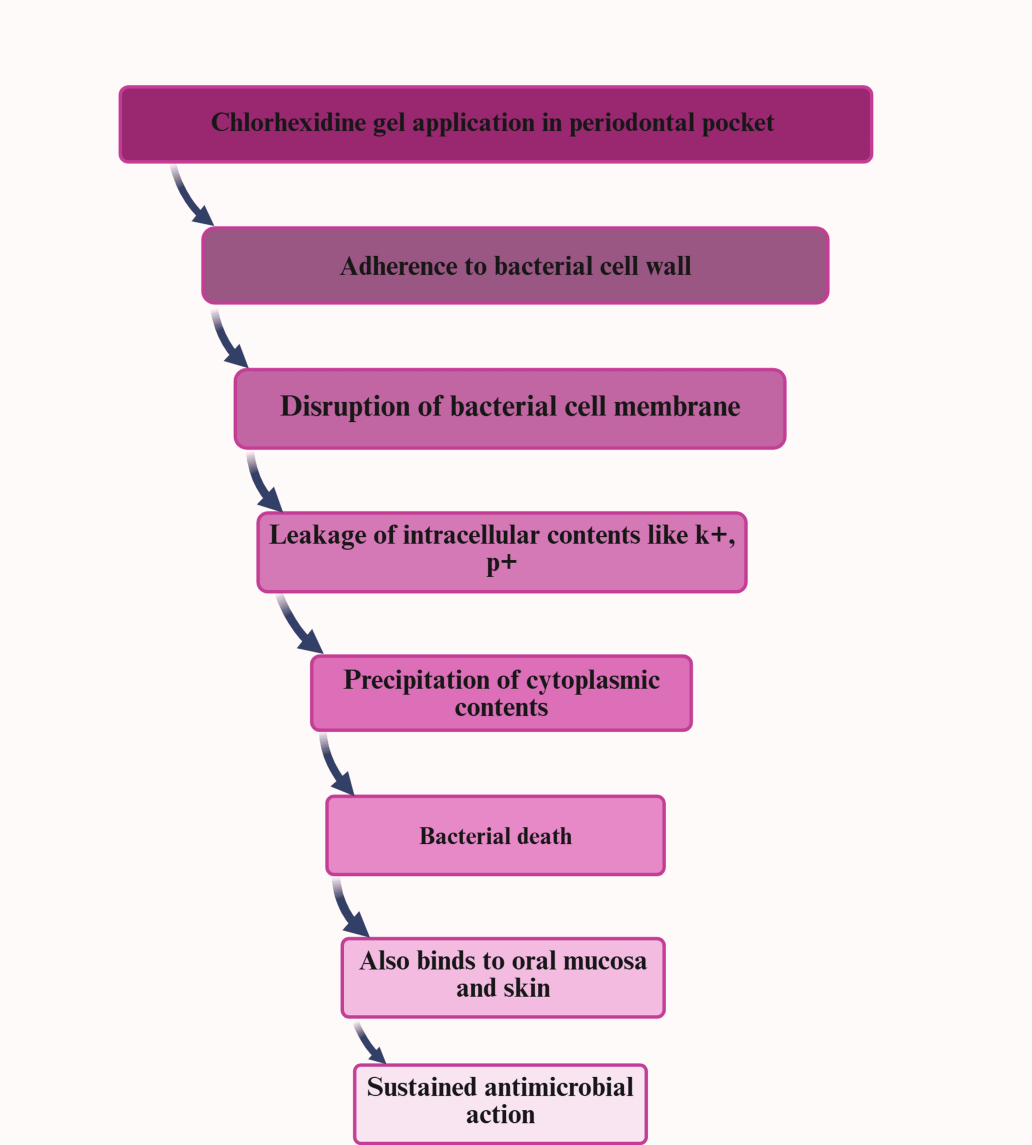
Mechanism of action of chlorhexidine. This figure was drawn using the premium version of BioRender, https://BioRender.com x4dlh7g, accessed on May 2, 2025, which has agreement number YX287TP91S [22]. Image Credit: Maitri Patel.

The effectiveness of adjunctive local CHX use alongside non-surgical periodontal therapy has been investigated using various pharmacological forms, yielding diverse clinical and microbiological results. Paolantonio et al. discovered significant reductions in PPD with CHX chips compared to SRP alone [25]. However, some studies did not observe substantial differences in PPD reduction or CAL gain. Moreover, the local application of CHX has been assessed in formulations such as xanthan gel [25]. Figure 6 illustrates the mechanism of action of CHX.

**Figure 7:**
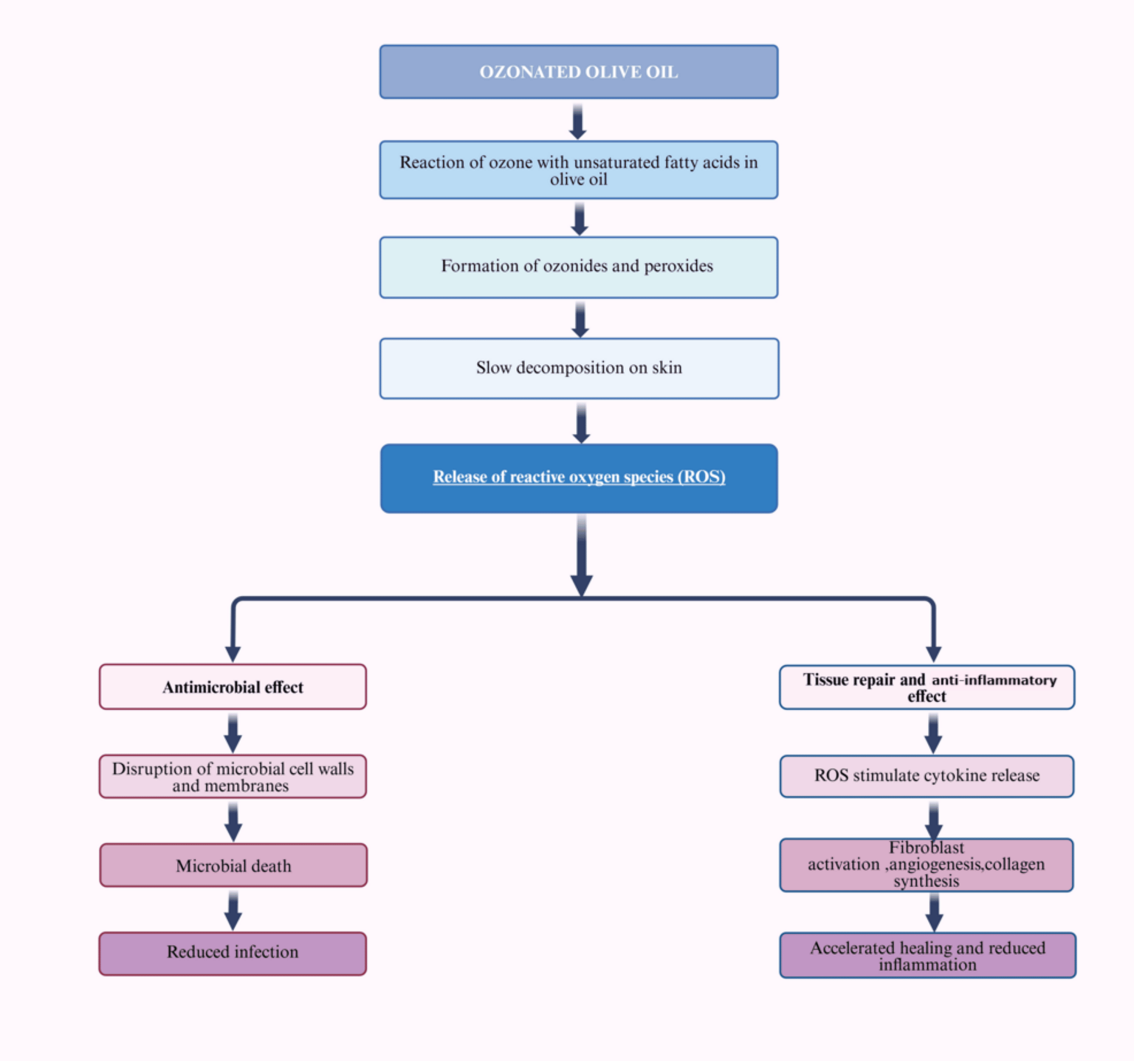
The mechanism of action of ozonated olive oil. This figure was drawn using the premium version of BioRender, https://BioRender.com x4dlh7g, accessed on May 2, 2025, which has agreement number CY287TPS1B [21]. Image Credit: Maitri Patel.

The research conducted by Eick et al. demonstrated considerable antibacterial effects of ozone against potential periodontal pathogens in vitro [12]. These results indicate that ozone has the potential as a supplementary treatment to SRP for individuals diagnosed with periodontal disease. Huth et al. [26] observed a reduction in periodontal pathogens with ozone irrigation compared to 0.2% CHX, except for *Aggregatibacter actinomycetemcomitans*. Additionally, Patel et al. [27] reported that using ozonated olive oil gel alongside SRP for chronic periodontitis treatment significantly improved clinical and microbiological parameters compared to control groups [10,28]. Figure 7 shows the mechanism of action of ozonated olive oil.

Shoukheba and Ali studied the effects of subgingival ozonated olive oil gel on patients with advanced periodontitis [10]. Fifteen patients received oral hygiene instructions, SRP, and ozonated olive oil gel as part of the control group. The assessed clinical indicators included PI, the gingival index (GI), bleeding on probing (BOP), and CAL. Except for BOP, which improved for up to three months, ozone treatment improved clinical indicators for up to six months. The antimicrobial properties of ozone may lead to resolving inflammation in periodontal tissues by reducing the pathogenic bacterial count. Research by Nagayoshi et al. showed ozone's high effectiveness against oral microorganisms, with a significant reduction in the viability of specific bacteria when treated with 0.5 mg/L ozonated water [29]. This suggests that ozone could have antibacterial effects against gram-negative periodontopathogens like *Porphyromonas gingivalis*, *Treponema forsythia*, and *Treponema denticola*, which are linked to BOP [30].

Kshitish and Laxman conducted a split-mouth, randomized, double-blind crossover study [28]. In this study, opposing mouth halves received subgingival irrigation with either ozone or CHX at varying intervals. Data on microbiology and clinical conditions were examined from the first to the seventh day. When ozone irrigation is compared to CHX, PI (12%) and GI (29%) show greater percentage improvements.

**Figure 8:**
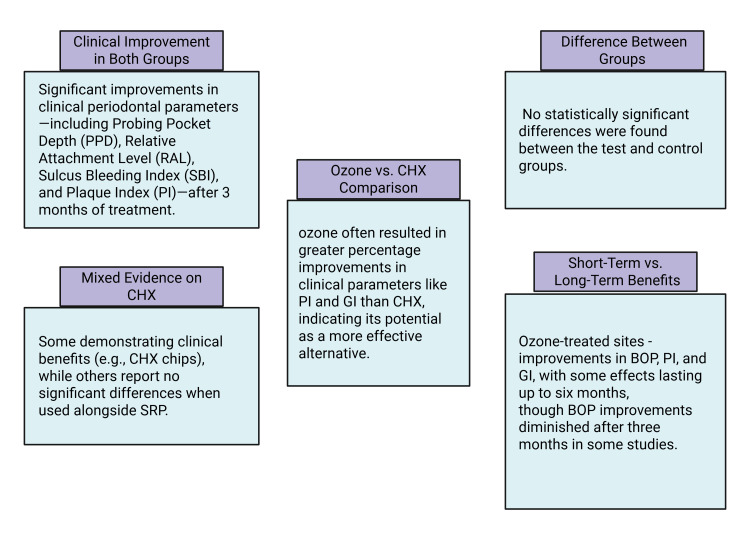
The principal findings of this paper. This figure was drawn using the premium version of BioRender, https://BioRender.com x4dlh7g, accessed on May 2, 2025, which has agreement number TL287TRS6U [22]. Image credit: Maitri Patel. CHX: chlorhexidine; SRP: scaling and root planing; GI: gingival index; BOP: bleeding on probing

Al Habashneh et al. investigated the clinical and biological impacts of incorporating ozone into non-surgical periodontal treatment [31]. At baseline and three months later, they measured several indicators, such as PI, BOP, GI, PPD, gingival recession, and CAL. Statistically significant enhancements were observed in all study parameters in both groups at baseline and three months, except GI [32], which must have been directly related to the anti-inflammatory effect of ozone. The principal findings of this paper are depicted in Figure 8.

Limitations of the study

This study has several limitations that should be considered when interpreting the results. The sample size was relatively small, which may limit the generalizability of the findings. Additionally, the short follow-up may not adequately reflect the periodontal disease's long-term efficacy or recurrence. Patient compliance with oral hygiene instructions could have influenced treatment outcomes. The lack of blinding may introduce observer or participant bias. Variability in the severity of chronic periodontitis among participants might have affected the comparability of treatment effects. This study did not include microbiological analysis to assess changes in pathogenic bacteria. This study did not include the assessment of inflammatory markers. Differences in the application techniques of ozonated olive oil and CHX gel may also have impacted the results. Lastly, systemic health factors that could influence periodontal healing were not fully accounted for.

Future recommendation

Future studies should include larger sample sizes and more extended follow-up periods to validate the findings and assess the long-term efficacy of ozonated olive oil in periodontal therapy. Advanced microbial and inflammatory biomarker analyses could provide deeper insights into its mechanisms of action. Exploring diverse formulations and combinations with other therapeutic agents may optimize its clinical application.

## Conclusions

This study suggests that ozonated olive oil and CHX gel are effective therapeutic agents in managing AP. Both treatments improved clinical parameters such as probing depth reduction and attachment level gain. Ozonated olive oil showed comparable efficacy to CHX gel, offering a potential natural alternative with fewer side effects. The findings highlight ozone therapy's antimicrobial and anti-inflammatory properties in periodontal care. However, individual patient responses may vary, and the treatment choice should consider patient tolerance and preferences. Further large-scale, long-term studies are needed to confirm these findings. Including microbiological assessments and evaluating systemic factors could enhance the understanding of their mechanisms. Overall, ozonated olive oil appears to be a promising adjunct in periodontal therapy.

## References

[1] Tonetti MS, Greenwell H, Kornman KS (2018). Staging and grading of periodontitis: framework and proposal of a new classification and case definition. J Periodontol.

[2] Nambiar S, Malothu S, Karmakar S, Varkey A, Chandra D, Chava VK (2022). Comparison of ozonated olive oil and chlorhexidine gel as an adjunct to nonsurgical periodontal therapy for the treatment of chronic periodontitis: a randomized controlled clinical trial. J Pharm Bioallied Sci.

[3] Scribante A, Gallo S, Pascadopoli M, Frani M, Butera A (2024). Ozonized gels vs chlorhexidine in non-surgical periodontal treatment: a randomized clinical trial. Oral Dis.

[4] Herrera D, Sanz M, Kebschull M (2022). Treatment of stage IV periodontitis: the EFP S3 level clinical practice guideline. J Clin Periodontol.

[5] Nagarakanti S, Gunupati S, Chava VK, Reddy BV (2015). Effectiveness of subgingival irrigation as an adjunct to scaling and root planing in the treatment of chronic periodontitis: a systematic review. J Clin Diagn Res.

[6] Matesanz-Pérez P, García-Gargallo M, Figuero E, Bascones-Martínez A, Sanz M, Herrera D (2013). A systematic review on the effects of local antimicrobials as adjuncts to subgingival debridement, compared with subgingival debridement alone, in the treatment of chronic periodontitis. J Clin Periodontol.

[7] Singh S, Roy S, Chumber SK (2009). Evaluation of two local drug delivery systems as adjuncts to mechanotherapy as compared to mechanotherapy alone in management of chronic periodontitis: a clinical, microbiological, and molecular study. J Indian Soc Periodontol.

[8] Zhao H, Hu J, Zhao L (2020). Adjunctive subgingival application of chlorhexidine gel in nonsurgical periodontal treatment for chronic periodontitis: a systematic review and meta-analysis. BMC Oral Health.

[9] Brookes ZL, Bescos R, Belfield LA, Ali K, Roberts A (2020). Current uses of chlorhexidine for management of oral disease: a narrative review. J Dent.

[10] Shoukheba MY, Ali SA (2014). The effects of subgingival application of ozonated olive oil gel in patient with localized aggressive periodontitis. A clinical and bacteriological study. Tanta Dent J.

[11] Huth KC, Saugel B, Jakob FM (2007). Effect of aqueous ozone on the NF-kappaB system. J Dent Res.

[12] Eick S, Tigan M, Sculean A (2012). Effect of ozone on periodontopathogenic species--an in vitro study. Clin Oral Investig.

[13] Indurkar MS, Verma R (2016). Effect of ozonated oil and chlorhexidine gel on plaque induced gingivitis: a randomized control clinical trial. J Indian Soc Periodontol.

[14] Singh A, S N, G CK, Pankaj C, Shweta D, Harshit S (2024). Effect of ozonated oil versus chlorhexidine gel among Indian patients with chronic periodontitis. Bioinformation.

[15] Feier R, Sireteanu Cucui RM, Ratiu RF (2023). Comparative study of ozonated olive oil and extra virgin olive oil effects on oral hygiene. Appl Sci.

[16] Caton JG, Armitage G, Berglundh T (2018). A new classification scheme for periodontal and peri-implant diseases and conditions - introduction and key changes from the 1999 classification. J Periodontol.

[17] Sanz M, Herrera D, Kebschull M (2020). Treatment of stage I-III periodontitis-the EFP S3 level clinical practice guideline. J Clin Periodontol.

[18] Aashik CR, Rajapandian K, Gayathri K, Ravishankar PL, Kalaivani V, Sunanda RK (2024). Evaluation of folic acid-containing mouthrinse and chlorhexidine mouthrinse as an adjunct to scaling and root planing in patients with periodontal disease. Cureus.

[19] Vandana KL, Gupta I (2009). The location of cemento enamel junction for CAL measurement: a clinical crisis. J Indian Soc Periodontol.

[20] Benamghar L, Penaud J, Kaminsky P, Abt F, Martin J (1982). Comparison of gingival index and sulcus bleeding index as indicators of periodontal status. Bull World Health Organ.

[21] Löe H (1967). The Gingival Index, the Plaque Index and the Retention Index Systems. J Periodontol.

[22] (2025). BioRender. https://app.biorender.com/illustrations/6813a95646d6d30a83735fa0..

[23] Gandhi KK, Cappetta EG, Pavaskar R (2019). Effectiveness of the adjunctive use of ozone and chlorhexidine in patients with chronic periodontitis. BDJ Open.

[24] Herrera D, Matesanz P, Bascones-Martínez A, Sanz M (2012). Local and systemic antimicrobial therapy in periodontics. J Evid Based Dent Pract.

[25] Paolantonio M, D'Angelo M, Grassi RF (2008). Clinical and microbiologic effects of subgingival controlled-release delivery of chlorhexidine chip in the treatment of periodontitis: a multicenter study. J Periodontol.

[26] Huth KC, Quirling M, Lenzke S (2011). Effectiveness of ozone against periodontal pathogenic microorganisms. Eur J Oral Sci.

[27] Patel PV, Patel A, Kumar S, Holmes JC (2012). Effect of subgingival application of topical ozonated olive oil in the treatment of chronic periodontitis: a randomized, controlled, double blind, clinical and microbiological study. Minerva Stomatol.

[28] Kshitish D, Laxman VK (2010). The use of ozonated water and 0.2% chlorhexidine in the treatment of periodontitis patients: a clinical and microbiologic study. Indian J Dent Res.

[29] Nagayoshi M, Fukuizumi T, Kitamura C, Yano J, Terashita M, Nishihara T (2004). Efficacy of ozone on survival and permeability of oral microorganisms. Oral Microbiol Immunol.

[30] Hrishi TS, Kundapur PP, Bhat GS, Vishwanath S, Kamath S (2020). Efficacy of subgingival ozone irrigation for management of chronic periodontitis - a clinical, microbiological and biochemical study. J Cont Med A Dent.

[31] Al Habashneh R, Alsalman W, Khader Y (2015). Ozone as an adjunct to conventional nonsurgical therapy in chronic periodontitis: a randomized controlled clinical trial. J Periodontal Res.

[32] Deepthi R, Bilichodmath S (2020). Ozone therapy in periodontics: a meta-analysis. Contemp Clin Dent.

